# COGNITIVE REAPPRAISAL AND STRESS REACTIVITY ACROSS ADULTHOOD

**DOI:** 10.1093/geroni/igae098.2702

**Published:** 2024-12-31

**Authors:** Jessica Maras, Kate Leger

**Affiliations:** University of Kentucky, Lexington, Kentucky, United States; University of Kentucky, Lexington, Kentucky, United States

## Abstract

How one reacts to stressful events, termed stress reactivity, impacts health and well-being. The use of emotion regulation strategies can influence reactivity, specifically cognitive reappraisal, which has been shown to reduce reactivity. Furthermore, age can affect reactivity due to older adults’ ability to employ cognitive reappraisal strategies more readily. The current study investigated the interplay between age and cognitive reappraisal on different components of reactivity: affective, somatic and physiological. Participants (N = 139) were divided into younger (N = 89) and older (N = 50) adults with subsets receiving the cognitive reappraisal intervention (N = 71) or the control condition (N = 68). Baseline measures were obtained via questionnaires followed by a stress-inducing task to assess reactivity. The cognitive reappraisal condition received a prompt prior to the stress-inducing task telling them to think about the positive aspects of the task. Reactivity was measured via negative affect, somatic symptoms (e.g., stomach ache) and blood pressure after the task, while controlling for baseline reactivity. Preliminary findings revealed a significant interaction between age and experimental condition for systolic blood pressure reactivity (β = -0.17, p =.049). This indicates that older adults in the control condition showed decreased systolic blood pressure reactivity to the stressor. No other significant main effects or interactions were found. This unexpected finding highlights that there may be specific age groups in which engaging in cognitive reappraisal is disadvantageous to different types of reactivity and emphasizes the need for understanding for whom and when cognitive reappraisal might work best.

